# The global energy balance as represented in CMIP6 climate models

**DOI:** 10.1007/s00382-020-05282-7

**Published:** 2020-05-25

**Authors:** Martin Wild

**Affiliations:** ETH Zurich, Institute for Atmospheric and Climate Science, 8001 Zurich, Switzerland

## Abstract

A plausible simulation of the global energy balance is a first-order requirement for a credible climate model. Here I investigate the representation of the global energy balance in 40 state-of-the-art global climate models participating in the Coupled Model Intercomparison Project phase 6 (CMIP6). In the CMIP6 multi-model mean, the magnitudes of the energy balance components are often in better agreement with recent reference estimates compared to earlier model generations on a global mean basis. However, the inter-model spread in the representation of many of the components remains substantial, often on the order of 10–20 Wm^−2^ globally, except for aspects of the shortwave clear-sky budgets, which are now more consistently simulated by the CMIP6 models. The substantial inter-model spread in the simulated global mean latent heat fluxes in the CMIP6 models, exceeding 20% (18 Wm^−2^), further implies also large discrepancies in their representation of the global water balance. From a historic perspective of model development over the past decades, the largest adjustments in the magnitudes of the simulated present-day global mean energy balance components occurred in the shortwave atmospheric clear-sky absorption and the surface downward longwave radiation. Both components were gradually adjusted upwards over several model generations, on the order of 10 Wm^−2^, to reach 73 and 344 Wm^−2^, respectively in the CMIP6 multi-model means. Thereby, CMIP6 has become the first model generation that largely remediates long-standing model deficiencies related to an overestimation in surface downward shortwave and compensational underestimation in downward longwave radiation in its multi-model mean.

## Introduction

The global energy balance fundamentally constrains the energy content of Earth’s climate system as well as its internal distribution. For more than a century, scientists have attempted to quantify the magnitudes of the components of the global energy balance (i.e., the energy balance averaged over the Earth’s sphere and over the year). Early attempts had to rely on a sparse number of observations taken at the surface and from balloon measurements combined with numerous assumptions, and the uncertainties in the global estimates were accordingly large (e.g., Abbot and Fowle 1908; Dines 1917). It was only with the advent of space-based measurements that the shortwave (solar) and longwave (thermal) energy exchanges between Earth and space could finally be quantified adequately, particularly through the Earth Radiation Budget Experiment (ERBE, Barkstrom et al. 1990) in the late 1980s and the more recent Clouds and Earth’s Radiant Energy System (CERES, Wielicki et al. 1996) mission since the beginning of the 2000s. These data have extensively been used for the assessment of the Top of Atmosphere (TOA) radiation budgets and cloud radiative effects in global climate models (GCMs) (e.g., Potter et al. 1992; Cess and Potter 1987; Potter and Cess 2004; Wild and Roeckner 2006; Trenberth and Fasullo 2010; Wang and Su 2013; Li et al. 2013; Dolinar et al. 2014). However, the distribution of the radiative energy within the climate system and at the Earth’s surface remained less well known also in the age of space-born measurements, since satellite measurements could provide only limited constraints on these aspects of the global energy balance. Thus, published estimates on the magnitudes of the global mean surface energy budget components still largely varied also in the satellite age, typically on the order of 10–20 Wm^−2^ or more (e.g., Ohmura and Gilgen 1993; Kiehl and Trenberth 1997; Wild et al. 1998, 2013; Hatzianastassiou et al. 2005; Trenberth et al. 2009; Stephens et al. 2012). Accordingly, throughout the history of model development, GCMs showed considerable discrepancies in their perception of the global energy balance, particularly at the Earth’s surface. The inter-model spread in the magnitudes of the individual components of the surface energy balance was known to be considerable since the earliest attempts of systematic model intercomparisons (Gutowski et al. 1991; Randall et al. 1992; Wild et al. 1995; Garratt and Prata 1996; Gleckler and Weare 1997; Li et al. 1997), whereas the agreement in their corresponding TOA components has been better. The latter was a consequence of the general practice to tune the GCMs to match their TOA flux magnitudes to the well-accepted space-born reference values, which became available since the late 1980s from ERBE and since the 2000s with even higher accuracy from CERES. No similar consensus reference values that could have served as tuning targets were available for the surface components, since these estimates historically showed large discrepancies as outlined above. However, with progress in the satellite-derived estimates of surface fluxes, as well as the availability of high accuracy radiation measurements from worldwide surface networks such as the Baseline Surface Radiation network (BSRN, Ohmura et al. 1998; Driemel et al. 2018), recent independently derived estimates of the global mean surface radiative components converged to within 4 Wm^−2^ (Wild 2017).

Comparisons with direct observations at the surface revealed a tendency of the GCMs to overestimate the downward shortwave radiation at the surface, and underestimate the downward longwave radiation, a long-standing problem that has persisted over several decades and generations of GCM development (Wild et al. 1995, 2013; Li et al. 1997; Cusack et al. 1998; Bodas-Salcedo et al. 2008; Wild 2008; Tang et al. 2019).

In the present study I will discuss the representation of the global energy balance in the latest generation of climate models participating in the sixth phase of the Coupled Model Intercomparison Project (CMIP6, Eyring et al. 2016), which will provide the basis for the upcoming Intergovernmental Panel on Climate Change (IPCC) 6th Assessment Report (AR6). The spatiotemporal focus will be on the global climatological annual mean, which will give a first order impression on the current model generations’ abilities to capture the overall energy distribution in the climate system. Their simulated global energy budgets will be intercompared and opposed to recently emerging reference estimates in the following. An adequate representation of the global mean energy budget provides a necessary, though not sufficient condition for a credible climate model.

## Data

At the time of the revision of this manuscript (March 2000), data from simulations performed by 40 GCMs appropriate for the present analysis have become available from CMIP6. Details on the modeling groups participating in CMIP6 can be found on the CMIP6 webpages of the Program for Climate Model Diagnosis and Intercomparison (PCMDI) (https://pcmdi.llnl.gov/CMIP6/).

The model-output variables under consideration for this study are the shortwave and longwave radiative fluxes at the surface and the TOA under both all-sky and clear-sky conditions, as well as the non-radiative fluxes of surface sensible and latent heat. They stem from the “historical all forcings” experiments of CMIP6, which aim at simulating the climate evolution since preindustrial times as realistic as possibly, considering all major natural and anthropogenic forcings, namely changes in solar output, atmospheric greenhouse gases, aerosol loadings (tropospheric and stratospheric volcanic), and land use (Eyring et al. 2016). These simulations cover the period 1850–2014. The global energy budgets of the CMIP6 models discussed in this study have been determined as averages over the final 15 years of these simulations (2000-2014) and shall represent present-day conditions at the beginning of the new millennium. To allow for a comparison with the previous model generation CMIP5 evaluated in Wild et al. (2013, 2015, 2019), I also determined the CMIP6 budgets for the averaging period 2000–2004 used in these former studies. The end year of 2004 was chosen in these studies since the corresponding historical simulation of the CMIP5 models only reached up to the year 2005 at the most. For the global mean budgets, the differences induced by the different averaging periods (2000–2014 versus 2000–2004) were, however, insignificant (< 0.3 Wm^−2^) for most components, with the exception of the longwave upward and downward radiation at the surface, which were enhanced by 0.6 and 0.8 Wm^−2^ in the 2000–2014 averaging period, due to the slightly stronger greenhouse forcing and associated warming. I further also investigated the interannual variability in the global annual mean energy budget components of the CMIP6 models, which turned out to be very small, with standard-deviations typically on the order of 0.2–0.3 Wm^−2^ for the global annual mean all-sky budget components, and even somewhat smaller for the respective clear-sky budgets. This further indicates that the exact length of the averaging period is not critical for the present analysis.

From many of the CMIP6 models, multiple realizations of the historic all forcings experiments with slightly differing initial conditions are available (ensemble simulations). The choice of the specific ensemble member is not critical, since their global multi-annual mean energy budgets do not differ significantly. Therefore, only one ensemble member from each model is included in the present analysis. Not all energy budget components were available from all models, therefore the number of models included in the analyses slightly varies depending on the energy balance component under investigation, as indicated in Table 1. The conclusions drawn in this study, however, were found to be very robust and do not critically depend on the exact number of models. The submitted version of this manuscript was based on a lower number of models available at the time (25 models), but the conclusions remained virtually identical in the present revised manuscript, despite the consideration of 50% additional models that became available in the meantime.

**Table 1:** Global annual mean estimates of the magnitudes of various energy balance components under clear-sky and all-sky conditions at the TOA, within the atmosphere and at the surface, representative for present-day climate

Energy balance component	Reference Estimates Wm^−2^	# CMIP6 models	CMIP6 mean Wm^−2^	CMIP6 spread Wm^−2^	CMIP6 stdev. Wm^−2^	CMIP5 mean Wm^−2^	CMIP5 spread Wm^−2^	CMIP5 stdev. Wm^−2^
**TOA**								
SW down TOA	340^a^, 340^b^, 340^c^	37	**340.2**	5.3	0.9	**341.3**	3.4	0.8
SW up all-sky TOA	− 99^a,^ − 100^b^, − 102^c^	38	**− 100.6**	13.1	2.7	**− 102.0**	12.6	3.1
SW absorbed all-sky TOA	241^a,^ 240^b^, 238^c^	37	239.5	14.5	2.9	239.2	11.2	3.0
SW up clear-sky TOA	− 53^a^, − 53^b^	37	− 53.0	7.7	1.9	− 52.6	11.2	2.3
SW absorbed clear-sky TOA	287^a^, 287^b^	37	**287.3**	7.1	1.8	**288.6**	10.6	2.1
SW CRE TOA	− 46^a^, − 47^b^	37	− 47.8	19.2	3.6	− 49.3	14.0	3.5
LW up (OLR) all-sky TOA	− 240^a^, − 239^b^, − 238^c^	40	− 238.3	15.6	2.8	− 238.0	11.7	2.9
LW up (OLR) clear-sky TOA	− 268^a^, − 267^b^	38	− 262.4	12.5	2.6	− 263.3	12.9	3.3
LW CRE TOA	28^a^, 28^b^	38	24.1	10.4	2.3	24.9	12.6	3.5
Net CRE TOA	− 18^a^, − 19^b^	37	− 23.6	13.5	3.3	− 24.1	15.5	3.9
Imbalance TOA	0.7^a^	37	1.1	4.5	0.8	1.2	n.a.	n.a.
**Atmosphere**								
SW absorbed all-sky atmos.	80^b^. 74^c^, 77^d^	37	**76.0**	8.9	2.0	**74.4**	9.9	2.8
SW absorbed clear-sky atmos.	73^b^, 73^d^	36	**72.8**	8.6	1.8	**70.1**	11.3	2.9
SW CRE atmos.	7^b^, 4^d^	36	**3.2**	4.0	1.1	**4.3**	8.8	1.6
LW net all-sky atmos.	− 183^b^, − 180^c^, − 187^d^	37	**− 182.1**	17.2	4.2	**− 179.8**	22.5	3.8
LW net clear-sky atmos.	− 183^b,^ − 184^d^	33	**− 180.9**	15.1	3.0	**− 179.1**	15.0	2.9
LW CRE atmos.	0^b^, − 3^d^	33	− 1.3	9.8	2.9	− 0.7	19.5	3.5
Net CRE atmos.	7^b^, 1^d^	33	**1.9**	10.0	2.6	**3.6**	18.9	4.1
**Surface**								
SW down all-sky surface	185 ^b^, 186 ^c^, 187^d^	38	**187.4**	20.8	4.5	**189.6**	15.8	4.7
SW up all-sky surface	− 25^b^, − 22^c^, − 23^d^	37	− 23.9	9.4	2.0	− 24.6	10.5	2.3
SW absorbed all-sky surface	160^b^, 164^c^, 164^d^	37	**163.4**	12.1	3.0	**165.0**	12.2	3.8
SW down clear-sky surface	247^b^, 244^d^	37	**244.8**	15.4	2.8	**249.7**	13.3	3.6
SW up clear-sky surface	33^b^, 30^d^	36	30.2	12.7	2.3	31.1	12.8	2.9
SW absorbed clear-sky surface	214^b^, 214^d^	36	**214.6**	11.0	2.2	**218.5**	15.5	3.6
SW CRE surface	− 54^b^, − 50^d^	36	**− 51.2**	20.4	4.0	**− 53.5**	16.7	4.1
LW down all-sky surface	342^b^, 341^c^, 344^d^	38	**343.8**	20.3	5.2	**340.1**	18.5	4.3
LW up all-/clear-sky surface	398^b^, 399^c^, 398^d^	37	− 399.9	11.7	3.0	− 398.7	10.7	2.6
LW net all-sky surface	− 56^b^, − 58^c^, − 54^d^	37	**− 56.2**	14.0	3.6	**− 58.6**	15.7	3.2
LW down clear-sky surface	314^b^, 314^d^	33	**318.0**	22.5	5.1	**314.5**	25.8	5.5
LW net clear-sky surface	− 84^b^, − 84^d^	33	**− 81.7**	16.1	3.5	**− 83.9**	15.9	3.7
LW CRE surface	28^b^, 30^d^	33	25.5	7.5	2.2	25.3	13.3	3.3
Net CRE surface	− 26^b^,− 20^d^	33	**− 25.4**	15.3	3.6	**− 28.2**	24.4	4.4
Net radiation surface	104^b^, 106^c^, 110^d^	37	107.2	13.1	3.1	106.2	17.2	3.9
Latent heat flux	− 82^b^, − 81^c^	38	− 85.3	18.0	3.5	− 85.8	13.9	3.9
Sensible heat flux	− 21^b^, − 25^c^	39	**− 20.1**	13.2	2.7	**− 18.9**	13.1	2.6
Surface Imbalance	0.6^b^, 0.5^c^	36	1.5	1.2	0.3	1.5	n.a.	n.a.

Given are recent reference estimates, together with the CMIP6 and CMIP5 model-calculated estimates in terms of their multi-model means, their inter-model spreads as well as their standard deviations

CMIP6 results from present study, CMIP5 results from Wild et al. (2019)

Units Wm^−2^

Reference estimates from Loeb et al. (2018) (^a^), Wild et al. (2015, 2019) (^b^), L’Ecuyer et al. (2015) (^c^) and Kato et al. (2018) (^d^)

Bold values indicate CMIP6 and CMIP5 multi-model means which are significantly different at the 95% confidence level

The reference values for the magnitudes of the TOA components stem from the Energy Balanced and Filled (EBAF) data set Edition 4.0 for the period 2001–2010 that resulted from the CERES mission (Loeb et al. 2018). In this mission, filtered radiances in the shortwave (between 0.3 and 5 μm), total (0.3 and 200 μm), and window (8 and 12 μm) regions are measured on board of the NASA satellites Terra and Aqua, with longwave radiances determined as differences between total and shortwave channel radiances. The uncertainty of the outgoing longwave flux at the TOA as measured by CERES due to the uncertainty in calibration is ~ 3.7 W m^−2^ (2 σ), whereas the uncertainty in the shortwave reflected flux is ~ 2% (2 σ), or equivalently 2 Wm^−2^ (Loeb et al. 2009). The CERES EBAF data set is gap-filled and adjusts the shortwave and longwave TOA fluxes within their range of uncertainty to be consistent with independent estimates of the global heating rate based upon in situ ocean observations (Loeb et al. 2018).

As references for the surface components, I use a number of recent estimates which are derived by independent approaches. Kato et al. (2018) developed an algorithm that forces computed TOA fluxes to match with the abovementioned CERES-EBAF TOA fluxes by adjusting surface, cloud, and atmospheric properties. Surface irradiances as provided in the CERES-EBAF surface product are subsequently adjusted using radiative kernels. L’Ecuyer et al. (2015) made use of a variety of satellite-derived products, and reintroduced energy and water cycle closure information lost in the development of these independently derived products through a variational method that explicitly accounts for the relative accuracies in all component fluxes. Wild et al. (2013, 2015, 2019) made use of the information contained in the direct flux measurements taken at worldwide surface observation sites and took into account the associated bias structure of a large number of GCMs to infer best estimates for the magnitudes of the global mean surface energy balance components. After decades of large discrepancies in published reference estimates for the global surface energy budget components, the abovementioned recent independent approaches provide estimates that converge to within a few Wm^−2^ on a global mean basis (Wild 2017). This increases the confidence in these references and enhances their usefulness as guidance in the assessment of the CMIP6 global mean energy budget components as discussed in the following.

## Results—all-sky budgets

### Shortwave components

**Fig. 1:**
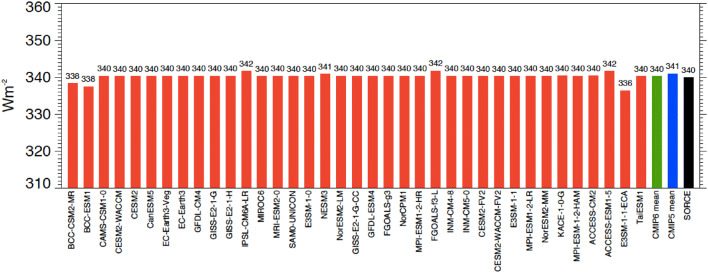
Global annual mean incoming shortwave radiation at the TOA as simulated by 37 individual CMIP6 models (red bars), by the CMIP6 multi-model mean (green bar), and the CMIP5 multi-model mean (blue bar). Reference estimate from the NASA Solar Radiation and Climate Experiment (SORCE, Kopp and Lean 2011) (black bar). Values can be multiplied by a factor of four to infer the solar constants used in the CMIP6 models. Units Wm^−2^

The global annual mean incoming shortwave radiation at the TOA in 37 CMIP6 models is shown in Fig. 1, with the quantification of the associated multi-model mean, range and standard deviation of model estimates given in Table 1. It is evident, that most models use a solar constant near 1361 Wm^−2^ (four times the values presented in Fig. 1, which represent the incoming shortwave radiation at the TOA per square meter on the Earth’s sphere, whereas the solar constant relates to the same quantity but per square meter on the cross-section of the Earth’s sphere). This is consistent with current best estimates from space-based observations of 1361 Wm^−2^ (Kopp and Lean 2011) provided by the NASA Solar Radiation and Climate Experiment (SORCE). There remain, however, a few models which still use a solar constant that deviates substantially from the 1361 Wm^−2^. The highest global mean incoming shortwave radiation at the TOA used in a CMIP6 model corresponds to a solar constant of 1367 Wm^−2^, the lowest to 1346 Wm^−2^. It is further interesting to note from Table 1 that the multi-model mean incoming shortwave radiation at the TOA is lower by 0.9 Wm^−2^ in CMIP6 than in the preceding model generation CMIP5 also presented in Table 1. This signifies that on average the solar constant used in the CMIP6 models is lower by 3.6 Wm^−2^ than in CMIP5 (again considering a factor of four), enforced by the developments in the measurement technologies that accounted for a lower value of the solar constant (Kopp and Lean 2011). Note that the difference in the multi-model mean estimates of the incoming shortwave radiation at the TOA in CMIP6 and CMIP5 is statistically significant at the 95% confidence level, as denoted by bold values in Table 1. The statistical significance at the 95% level of the differences between the CMIP5 and CMIP6 multi-model means in Table 1 has been determined by gaussian error propagation rules from the standard deviations of the individual models in CMIP5 and CMIP6.

**Fig. 2:**
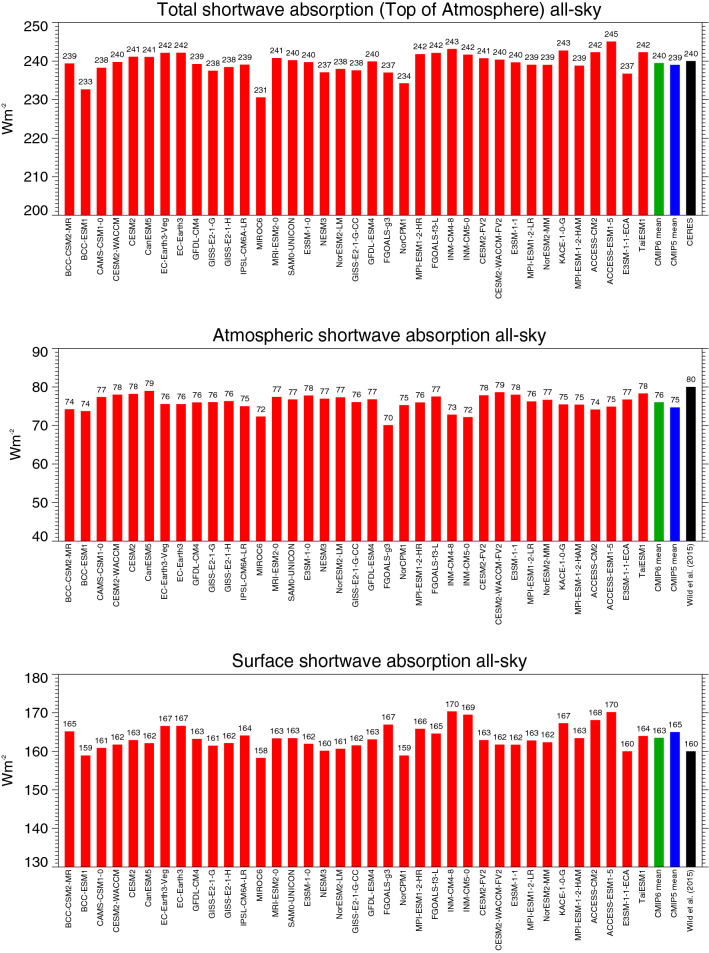
Global annual mean shortwave all-sky radiation budgets representative for present-day climate. Shortwave radiation absorbed at the surface (lower panel), within the atmosphere (middle panel), and in the total climate system (TOA, upper panel), as simulated by 37 individual CMIP6 models (red bars). CMIP6 and CMIP5 multi-model means given by green and blue bars, respectively. Reference estimates from CERES (Loeb et al. 2018) and Wild et al. (2015) (black bars). Units Wm^−2^

The global annual mean shortwave absorption in the total climate system (TOA), within the atmosphere and at the Earth’s surface of 37 CMIP6 climate models is shown in Fig. 2, with the statistical summary given in Table 1. The individual models vary in their simulated global mean shortwave budgets with standard deviations near 3 Wm^−2^ both at the TOA and the surface (Table 1). Table 1 further shows that the inter-model spread in these budgets in the CMIP6 models is as large as in the preceding model generation CMIP5, despite the slightly lower number of CMIP6 models providing the shortwave budgets (37 models) compared to CMIP5 (43 models, Wild et al. 2015).

Compared to the reference values, the multi-model mean TOA shortwave absorption, at 239.5 Wm^−2^ globally, closely matches the satellite-based reference estimates near 240 ± 2 Wm^−2^ (Table 1). This is favored by the fact that the various modelling groups aim at tuning their TOA energy fluxes to match the CERES-EBAF reference estimates on a global mean basis. Individual models, however, still differ by up to 9 Wm^−2^ from these reference estimates (Fig. 2). Given the tuning efforts undertaken by all modelling groups, this is surprising, as well as the fact that 9 out of 37 CMIP6 models simulate a TOA shortwave absorption outside the 2-sigma observational uncertainty ranges (± 2 Wm^−2^) of the CERES reference values (tuning targets) given in Loeb et al. (2009).

**Fig. 3:**
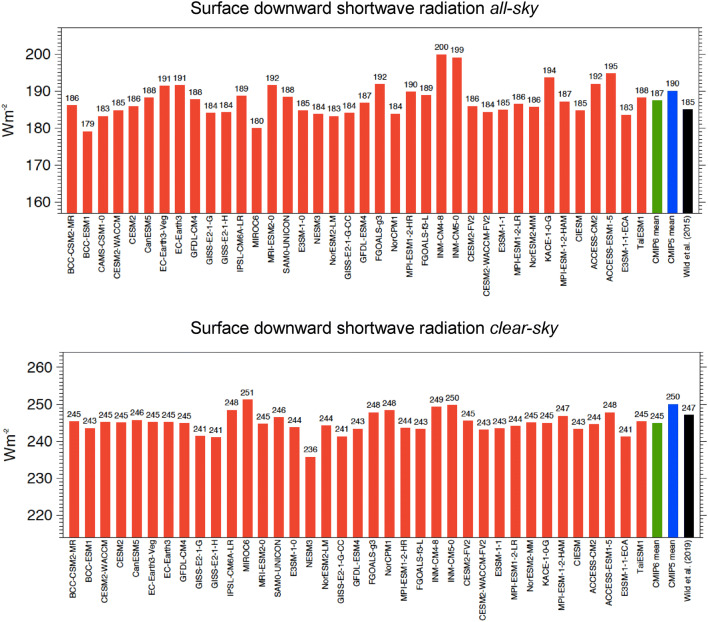
Global annual mean downward shortwave radiation at Earth’s surface representative for present-day climate under all-sky (upper panel) and clear-sky conditions (lower panel), as simulated by various CMIP6 models (red bars). CMIP6 and CMIP5 multi-model means given by green and blue bars, respectively. All-sky and clear-sky reference estimates from Wild et al. (2015, 2019), respectively (black bars). Clear-sky fluxes determined using Method II according to Cess and Potter (1987). Units Wm^−2^

Also at the surface, the multi-model mean shortwave absorption is, at 163.4 Wm^−2^ globally, close to recent reference estimates of 160–164 Wm^−2^ (Wild et al. 2015; L’Ecuyer et al. 2015; Kato et al. 2018), again with substantial deviations by some individual models. Still, two-thirds of the model-calculated estimates fall within the range given by the above references. The global multi-model mean surface shortwave absorption in CMIP6 is lower by 1.6 Wm^−2^ than in CMIP5 (165 Wm^−2^) (statistically significant, Table 1). The lower multi-model mean absorption at the surface in CMIP6 is mostly due to a somewhat higher atmospheric shortwave absorption. The global multi-model mean atmospheric shortwave absorption in CMIP6 amounts to 76.0 Wm^−2^, compared to the corresponding value of 74.4 Wm^−2^ in CMIP5 (difference statistically significant, Table 1). The higher atmospheric absorption in CMIP6 leads also to a global mean downward shortwave radiation at the Earth’s surface, which is, at 187.4 Wm^−2^, lower by more than 2 Wm^−2^ compared to CMIP5 (statistically significant, Table 1), and thereby in closer agreement with recent reference estimates (Table 1). But note also the large spread in the global mean downward shortwave radiation at the Earth’s surface amongst the various CMIP6 models in Fig. 3 (upper panel), which amounts to as much as 21 Wm^−2^. This spread is more than 8 Wm^−2^ larger than the spread in the corresponding surface absorbed shortwave radiation (Table 1). This implies that the surface albedos in some of the CMIP6 models partly compensate for the discrepancies in the simulated incoming shortwave radiation at the Earth’s surface, with a tendency for higher and lower surface albedos in models with high and low incoming shortwave radiation, respectively (correlation coefficient 0.73).

### Longwave components

**Fig. 4:**
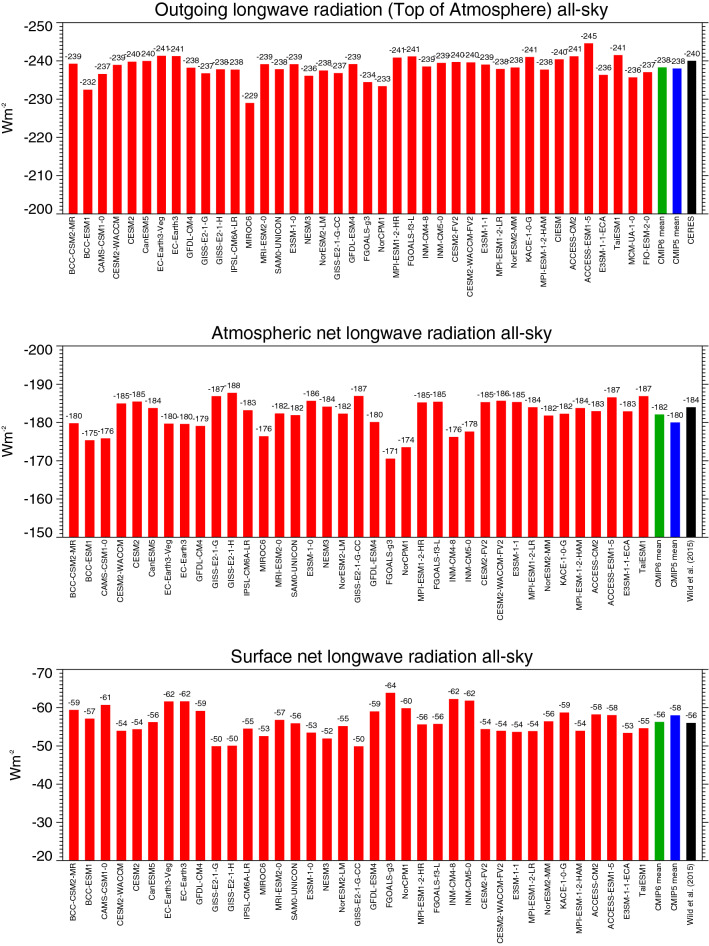
Global annual mean longwave all-sky radiation budgets representative for present-day climate. Net longwave radiation at the surface (lower panel), within the atmosphere (middle panel), and emitted to space (upper panel) as simulated by various CMIP6 models (red bars). CMIP6 and CMIP5 multi-model means given by green and blue bars, respectively. Reference estimates from CERES (Loeb et al. 2018) and Wild et al. (2015) (black bars). Units Wm^−2^

Global annual mean estimates of the net longwave radiation at the TOA (outgoing longwave radiation, OLR), within the atmosphere and at the surface as simulated by the various CMIP6 models are shown in Fig. 4. The spread amongst the models amounts to 15.6, 17.2, and 14.0 Wm^−2^, with standard deviations of 2.8, 4.2 and 3.6 Wm^−2^ for the OLR, the net atmosphere and net surface longwave radiation, respectively (Table 1). As for the shortwave budgets discussed above, also for the longwave budgets of the CMIP6 models this implies no convergence in their individual estimates compared to CMIP5 (Table 1). The inter-model spread in the simulated global mean OLR is even considerably larger in CMIP6 than in CMIP5, and also in terms of standard deviations, the CMIP6 models differ as much or more in their longwave budgets as their CMIP5 counterparts. In terms of absolute magnitudes, the CMIP6 multi-model mean, at 238.3 Wm^−2^ nearly matches the CMIP5 multi-model mean estimate, and is close to the satellite-based reference values of 240 ± 3 Wm^−2^ (Table 1). This is again largely a reflection of the tuning of the models to match the CERES values. Still, individual CMIP6 models do deviate by up to 11 Wm^−2^ from this reference value (Fig. 4, upper panel). Specifically, 8 out of 40 CMIP6 models simulate a global mean OLR outside the 2-sigma observational uncertainty given in Loeb et al. (2009) for the CERES reference value.

**Fig. 5:**
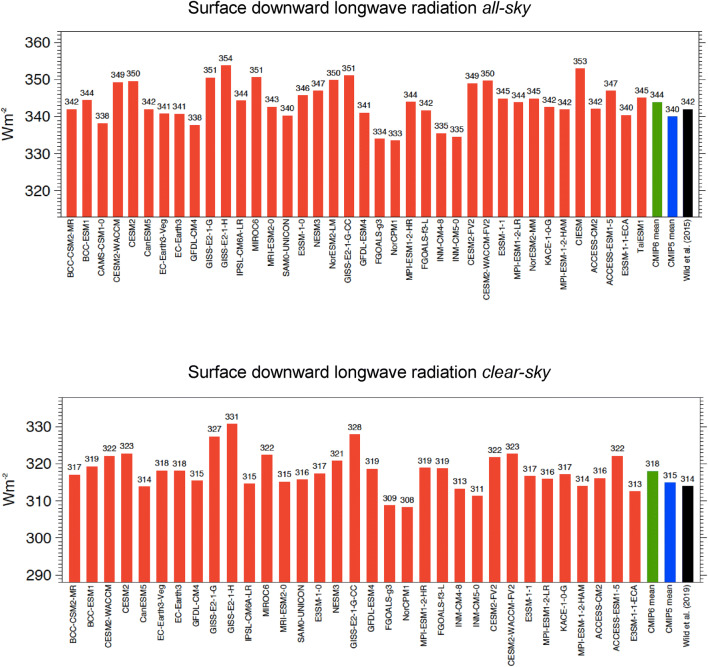
Global annual mean downward longwave radiation at Earth’s surface for present-day climate under all-sky (upper panel) and clear-sky conditions (lower panel), as simulated by various CMIP6 models (red bars). CMIP6 and CMIP5 multi-model means given by green and blue bars, respectively. All-sky and clear-sky reference estimates from Wild et al. (2015, 2019), respectively (black bars). Clear-sky fluxes are determined using Method II according to Cess and Potter (1987). Units Wm^−2^

The global mean net surface longwave budget in the multi-model mean in CMIP6 is, at − 56.2 Wm^−2^, more than 2 Wm^−2^ less negative than in CMIP5 (− 58.6 Wm^−2^) (statistically significant, Table 1), i.e. the surface longwave cooling in CMIP6 is less effective than in the CMIP5 multi-model mean (Table 1). This is largely caused by a 3.7 Wm^−2^ higher surface downward longwave radiation in the CMIP6 multi-model mean compared to CMIP5 (statistically significant, Table 1), which is not compensated by the 1.2 Wm^−2^ higher multi-model mean surface upward longwave radiation in CMIP6 (Table 1). The higher global mean downward longwave radiation in the CMIP6 models, at 343.8 Wm^−2^ in the multi-model mean comes now very close to the reference estimates given in Tables 1 and 3 (see discussion in Sect. 6). Yet note that, similarly to the downward shortwave radiation (Sect. 3.1), the spread in the global mean downward longwave radiation amongst the individual CMIP6 models remains considerable, covering as much as 20 Wm^−2^ (Fig. 5, upper panel, Table 1).

### Net radiation balance and non-radiative fluxes

**Fig. 6:**
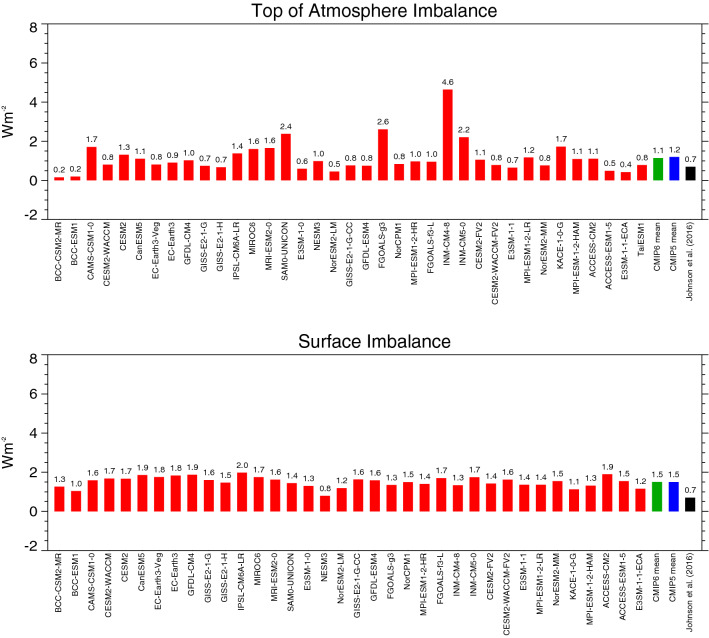
Global annual mean energy imbalance at the TOA (upper panel) and at the Earth’s surface (lower panel) for present-day conditions as simulated by various CMIP6 models (red bars). CMIP6 and CMIP5 multi-model means given by green and blue bars, respectively. Reference estimates from Johnson et al. (2016) (black bars). TOA energy imbalance determined as difference between absorbed shortwave radiation in the climate system (Fig. 2, upper panel) and the longwave emission to space (Fig. 4, upper panel). Surface imbalance determined as difference between surface net radiation (Fig. 7, upper panel) and the sum of surface sensible and latent heat fluxes (Fig. 7, middle/lower panels). Units Wm^−2^

If the Earth’s climate system is in equilibrium, the shortwave radiation absorbed by the climate system should match the outgoing longwave radiation at the TOA on a global annual mean basis. Currently, with anthropogenic climate change, the climate system is slightly out of balance, with less longwave radiation emitted out to space than absorbed by our planet, so that energy is accumulating in the climate system, leading to global warming (Hansen et al. 2005). This imbalance is estimated to be slighly less than 1 Wm^−2^ on a global mean basis, based on measurements of changes in the heat content of the oceans (Hansen et al. 2005; von Schuckmann et al. 2016; Johnson et al. 2016). These measurements stem from a global array of more than 4000 free-drifting profiling floats, known as ARGO, that record the temperature and salinity of the upper 2000 m of the oceans since the early 2000s, which allows for the first time a continuous monitoring of the change in the energy content in the oceans. Since more than 90% of the energy accumulation induced by the TOA radiation imbalance is stored in the world’s oceans due to their large heat capacities, their change in the energy content is considered a good measure of the radiative imbalance at the TOA (e.g., Hansen et al. 2005; von Schuckmann et al. 2016; Johnson et al. 2016). Most of the CMIP6 models show a positive TOA imbalance of different magnitudes over the averaging period 2000-2014 considered here, with a multi-model mean of 1.1 Wm^−2^ not too far away from the reference estimates, such as the 0.7 Wm^−2^ given by Johnson et al. (2016) (Fig. 6, upper panel). Since energy might not be 100% preserved in some of the numerical schemes used in the climate models (Hourdin et al. 2017), not too much weight should be placed on the exact magnitudes of these simulated values. While most models show imbalances reasonably close to the reference estimates, the imbalances cover still a range of more than 4 Wm^−2^, and some of the models show unrealistically high imbalances, pointing to problems in energy conservation in these models.

The surface net radiation (also known as surface radiation balance) consists of the absorbed shortwave radiation and the net longwave cooling at the Earth’s surface. It provides the energy available for the non-radiative fluxes of the surface energy balance, particularly the surface sensible and latent heat fluxes.

**Fig. 7:**
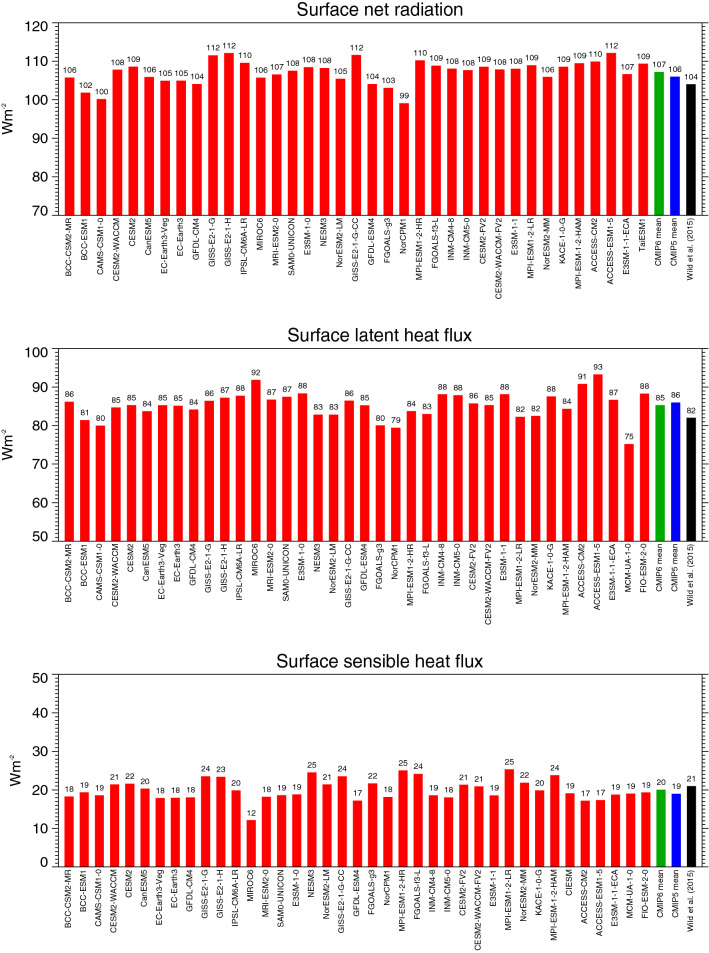
Global annual mean surface net radiation (upper panel), latent heat fluxes (middle panel) and sensible heat fluxes (lower panel) representative for present-day climate as calculated by various CMIP6 models (red bars). CMIP6 and CMIP5 multi-model means given by green and blue bars, respectively. Reference estimates from Wild et al. (2015) (black bars). Units Wm^−2^

The global mean surface net radiation in the various CMIP6 models is shown in Fig. 7 (upper panel), together with their global mean latent (middle panel) and sensible heat fluxes (lower panel). The globally averaged surface net radiation in the CMIP6 models is, at 107.2 Wm^−2^, slightly higher than the corresponding value of CMIP5 (106.2 Wm^−2^). However, compared to CMIP5, the CMIP6 multi-model mean estimate is composed of a lower surface shortwave absorption, which is overcompensated by a lower surface net longwave cooling due to the higher downward longwave radiation. The surface net radiation in the CMIP6 global multi-model mean is still somewhat higher than the estimates provided by Wild et al. (2015) and L’Ecuyer et al. (2015) (Table 1). The spread and standard deviation in the global mean surface net radiation amongst the 37 individual CMIP6 models is, with 13 Wm^−2^ and 3.1 Wm^−2^ respectively, also still substantial, but somewhat smaller than in CMIP5.

The latent heat flux is an interesting quantity, since it makes the link between the global energy and water balance. The latent heat flux is the energy equivalent of evaporation, which in the global annual mean equals precipitation. Thus, differences in the magnitudes of the global mean latent heat flux in the various models reflect also differences in global evaporation and precipitation, and therefore in the intensity of the global water cycle. The multi-model mean latent heat flux is, at 85.3 Wm^−2^, slightly above the recently published reference estimates (Table 1). Reference estimates for the global mean latent heat flux can be inferred from observational-based global precipitation estimates. However, these estimates are still afflicted with considerable uncertainties.

The individual CMIP6 models on the other hand differ in their simulated global mean latent heat fluxes by up to 18 Wm^−2^, which corresponds to a spread of as much as 21%, considering the multi-model mean latent heat flux of 85 Wm^−2^ (Fig. 7, middle panel). This implies that the simulated global mean precipitation between the individual CMIP6 models also must have the same spread of 21%, or, in other words, the intensity of the global water cycle simulated by the different CMIP6 models varies in range of more than 20%). This is even larger than amongst the 43 CMIP5 models, where the intensity of the water cycle in terms of their global latent heat fluxes varied in a range of 16% (14 Wm^−2^) (Table 1). Thus, there is no indication that the considerable discrepancies in the quantitative representation of the global water cycle in the various models reduce in CMIP6.

The global mean sensible heat flux is poorly constrained from an observational perspective. The CMIP6 models, with a multi-model mean sensible heat flux of 20.1 Wm^−2^ globally, are close to the estimate in Wild et al. (2015) of 21 Wm^−2^ as well as related estimates from reanalyses (Trenberth et al. 2009; Wild et al. 2013 and references therein), yet somewhat lower than the estimates given in Stephens et al. (2012) and L’Ecuyer et al. (2015) (Table 1). However, the global mean sensible heat fluxes in individual CMIP6 models vary in a range of 13 Wm^−2^, which corresponds to a spread of as much as 65% (Fig. 7, lower panel, Table 1). This wide spread reflects the considerable uncertainties still inherent in the quantification of the sensible heat fluxes in climate models.

In addition, the global annual mean energy imbalance at the Earth’s surface of the CMIP6 models is shown in Fig. 6 (lower panel), which refers to the difference between the surface net radiation and the sum of the surface sensible and latent heat fluxes, and which is closely related to the TOA energy imbalance discussed above. Most of this energy goes into the oceans, while a small fraction is stored in the terrestrial sub-surfaces and used for the melting of snow and ice. All models show a positive surface imbalance as expected with increasing greenhouse-gas forcing, with values mostly between 1 and 2 Wm^−2^, and a multi-model mean of 1.5 Wm^−2^ (Table 1, Fig. 6, lower panel). This is slightly higher than the reference values which are somewhat below 1 Wm^−2^ (Hansen et al. 2005; von Schuckmann et al. 2016; Johnson et al. 2016), again potentially due to imperfect energy conservation in the models (Hourdin et al. 2017). The potential lack of precise energy conservation in the individual models may also be the reason that the TOA and surface imbalances are not obviously correlated across models.

## Results—clear-sky budgets

### Shortwave components

**Fig. 8:**
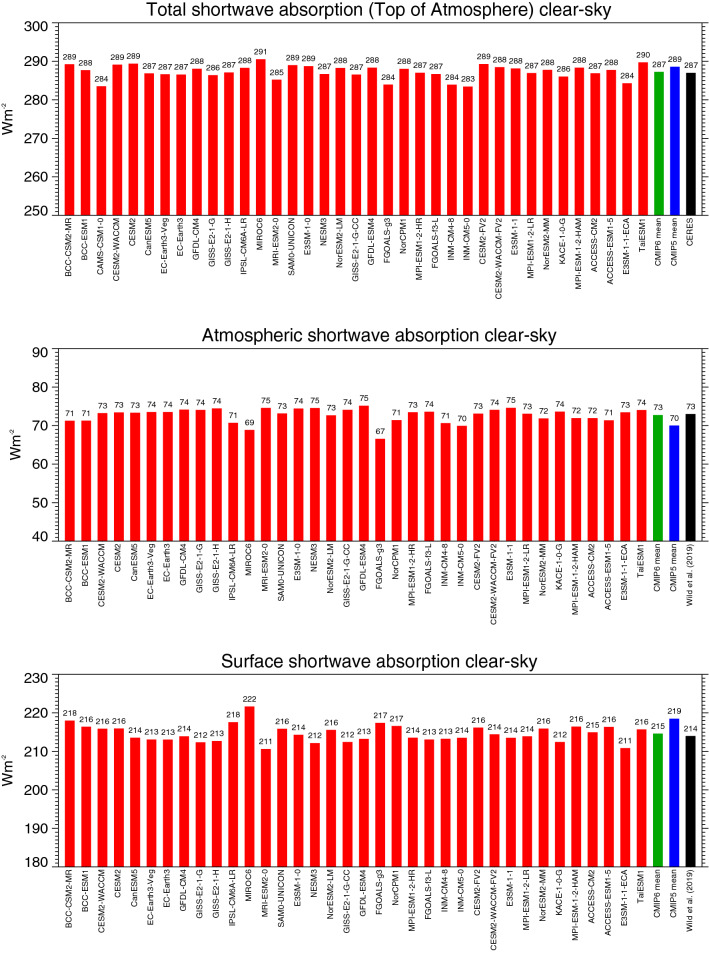
Global annual mean shortwave clear-sky radiation budgets representative for present-day climate. Shortwave clear-sky radiation absorbed at the surface (lower panel), within the atmosphere (middle panel), and in the total climate system (TOA, upper panel) as simulated by various CMIP6 models (red bars). CMIP6 and CMIP5 multi-model means given by green and blue bars, respectively. Reference estimates from CERES (Loeb et al. 2018) and Wild et al. (2019) (black bars). Units Wm^−2^

Shown in Fig. 8 are the global annual mean shortwave budgets in the absence of clouds (“clear-sky”) of various CMIP6 models at the TOA (upper panel), within the atmosphere (middle panel) and at the surface (lower panel). The cloud-free fluxes in the climate models are determined according to the so-called “Method II” (Cess and Potter 1987; Potter et al. 1992), i.e. the clear-sky fluxes are determined at every model-timestep, irrespective of the presence or absence of clouds. Thus, clear-sky fluxes are also calculated during cloudy conditions in the models, just by removing the clouds in the radiative transfer calculations, but otherwise retaining the atmospheric conditions prevailing during these cloudy conditions. Observational reference estimates which consider only “true” cloud-free conditions (Method I according to Cess and Potter (1987), have therefore to be slightly adjusted to match the clear-sky definition as used in the model world (see Wild et al. 2019).

The shortwave clear-sky TOA budget determines the amount of shortwave radiation absorbed in the cloud-free climate system. In the CMIP6 global multi-model mean, this amounts to 287.3 Wm^−2^, which perfectly matches the observational reference value from CERES (Loeb et al. 2018), slightly adjusted to satisfy Method II as described in Wild et al. (2019) to account for the different clear-sky definitions in models and observations as outlined in the paragraph above. Again the agreement between simulated and observed fluxes is partly an outcome of the tuning process of the models. The CMIP6 multi-model mean clear-sky shortwave TOA absorption is somewhat smaller than in CMIP5 by 1.3 Wm^−2^, indicative of a slightly higher clear-sky planetary albedo in the CMIP6 multi-model mean (statistically significant, Table 1). The inter-model spread and standard deviation of the clear-sky shortwave TOA absorption amongst the CMIP6 models are almost half of the corresponding ones under all-sky conditions, as might be expected when the complicating cloud-effects are excluded in the flux calculations.

The absorption of shortwave radiation in the cloud-free atmosphere in the multi-model mean is, at 72.8 Wm^−2^ globally, higher by 2.7 Wm^−2^ than in the CMIP5 models (statistically significant, Table 1). This brings the CMIP6 multi-model mean in almost perfect match with the reference estimate of 73 Wm^−2^ determined in independent approaches by Wild et al. (2015) and Kato et al. (2018) (Table 1). It is noteworthy that not only the multi-model mean but also many individual models closely match the reference values of 73 Wm^−2^. 33 out of 36 models determine the atmospheric clear-sky shortwave absorption to within 2 Wm^−2^ from these reference values (Fig. 8, middle panel). This is even more remarkable, as this quantity has been notoriously underestimated over generations of GCMs, as further discussed in Sect. 6. The shortwave clear-sky budgets simulated in the various CMIP6 models are generally more consistent than in CMIP5, as evident in smaller spreads and standard deviations (Table 1). This is in contrast to most other components of the global energy balance which typically show no reduction in terms of inter-model spreads and standard deviations from CMIP5 to CMIP6.

The absorption of shortwave radiation at the Earth’s surface under cloud-free conditions is in the CMIP6 multi-model mean at 214.6 Wm^−2^ globally almost 4 Wm^−2^ lower than in CMIP5 (statistically significant, Table 1). This is primarily caused by the higher clear-sky shortwave atmospheric absorption (by 2.7 Wm^−2^), as well as by the slightly lower overall (net TOA) clear-sky shortwave absorption (by 1.3 Wm^−2^) as mentioned above and seen in Table 1. The CMIP6 multi-model mean clear-sky shortwave absorption is also in near perfect match with the two independently derived reference estimates of Kato et al. (2018) and Wild et al. (2019), both consistently at 214 Wm^−2^, and thus no longer indicates an overestimation as noted in the CMIP5 models (Table 1, Wild et al. 2019) and in previous model generations. Again it is remarkable, that 29 out of 36 CMIP6 models simulate a global mean clear-sky surface shortwave absorption that is within 2 Wm^−2^ of the above reference estimates (Fig. 8, lower panel).

The lower clear-sky surface shortwave absorption in the CMIP6 models is also in line with a substantially lower surface downward shortwave clear-sky radiation in these models, which is, at 244. 8 Wm^−2^ lower by almost 5 Wm^−2^ than in CMIP5 (statistically significant, Table 1). This lower surface downward shortwave clear-sky radiation in the CMIP6 multi-model mean leads then again to a better agreement with the reference estimates of Wild et al. (2019) and Kato et al. (2018) (Table 1).

Overall, the global mean shortwave radiation budget under cloud-free conditions in CMIP6 is in remarkable agreement with recent reference estimates, not only in its multi-model mean which is within 1 Wm^−2^ of the reference values for the total (TOA), atmosphere and surface absorption, but also in the majority of the individual models which are in close agreement with these references. This indicates a clear improvement compared to previous model generations in these quantities, and increases confidence both in the model-calculated and reference estimates of the shortwave clear-sky budgets.

### Longwave components

**Fig. 9:**
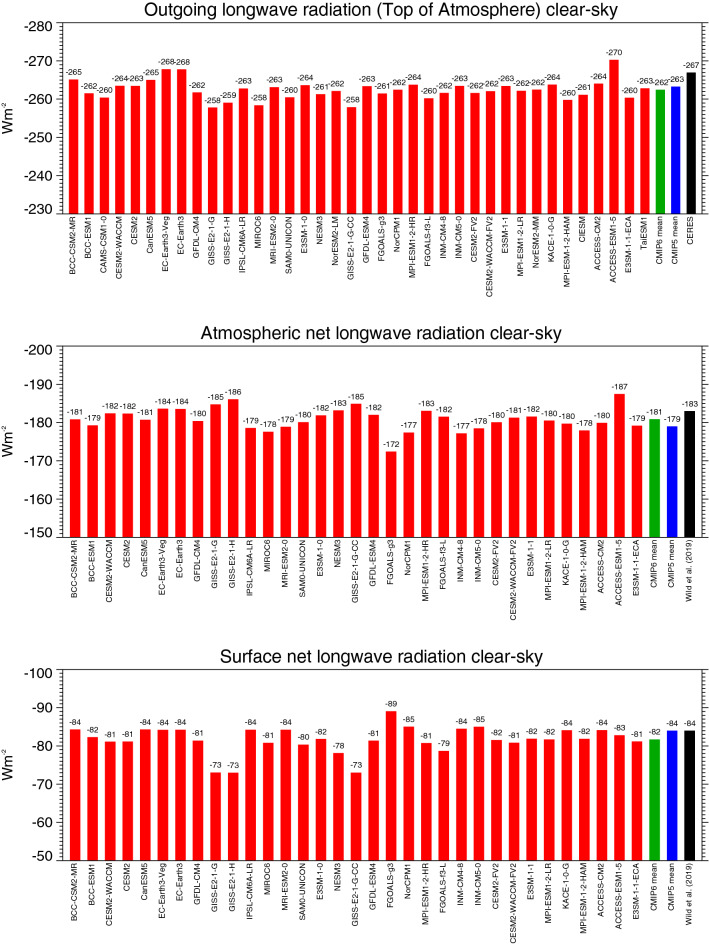
Global annual mean longwave clear-sky radiation budgets representative for present-day climate. Net clear-sky longwave radiation at the surface (lower panel), within the atmosphere (middle panel), and emitted to space (upper panel) as simulated by various CMIP6 models (red bars). CMIP6 and CMIP5 multi-model means given by green and blue bars, respectively. Reference estimates from CERES (Loeb et al. 2018) and Wild et al. (2019) (black bars). Units Wm^−2^

The global mean longwave budget under cloud-free conditions of the various CMIP6 models is presented in Fig. 9, with the clear-sky OLR in the upper panel, and the longwave clear-sky budget in the atmosphere and at the surface in the middle and lower panels, respectively.

The CMIP6 multi-model-mean clear-sky OLR is, at – 262.4 Wm^−2^ globally, lower by 1 Wm^−2^ compared to CMIP5. Quantitatively, both these amounts are a fair bit smaller than the latest CERES Ed 4.0 reference estimate (− 268 Wm^−2^, Loeb et al. 2018), slightly adjusted to − 267 Wm^−2^ to conform with Method II (Wild et al. 2019). As in CMIP5, the lower model values might have been favored by earlier CERES product releases (Ed 2.8 and Ed2 SYN1deg-Month) with somewhat smaller clear-sky OLR estimates, which may have been used as target estimates in the model tuning process.

The net longwave cooling of the cloud-free atmosphere is, at – 180.9 Wm^−2^, somewhat stronger in the CMIP6 multi-model mean than in CMIP5, particularly due to a stronger clear-sky emission towards the surface (clear-sky surface downward longwave radiation), which is higher by 3.5 Wm^−2^ in the global multi-model mean (statistically significant, Table 1). Accordingly, the global multi-model mean net longwave cooling at the Earth’s surface is weaker in CMIP6 compared to CMIP5 by 2.2 Wm^−2^, since the slightly higher surface longwave upward radiation in CMIP6 of 1.2 Wm^−2^ cannot compensate for the 3.5 Wm^−2^ additional energy that the surface obtains from the enhanced downward longwave clear-sky emission in CMIP6 (Table 1, Fig. 5, lower panel). The discrepancies amongst the simulated surface net longwave clear-sky budgets in the various CMIP6 models remain substantial (Fig. 9, lower panel), and are substantially larger both in terms of spread and standard deviation compared to their shortwave counterparts, i.e. the surface shortwave clear-sky absorption, despite their smaller absolute amounts (cf. Fig. 8 lower panel, Table 1).

In terms of absolute values, the downward longwave clear-sky radiation is, at 318.0 Wm^−2^ now larger than the independent reference estimates of Wild et al. (2019) and Kato et al. (2018), both at 314 Wm^−2^. Note also the particularly large spread in the downward longwave clear-sky radiation amongst the 37 CMIP6 models (22.5 Wm^−2^, Fig. 5 lower panel), which is thus the quantity with the largest spread of all CMIP6 energy balance components discussed in this study. This already applied for the CMIP5 models (Wild et al. 2019). Also, as in CMIP5 and in earlier model intercomparison projects, the spread amongst the simulated global mean downward longwave clear-sky radiation in the various CMIP6 models is larger (22.5 Wm^−2^) than in their all-sky counterparts (20.3 Wm^−2^) (Fig. 5 and Table 1). This confirms findings based on earlier model generations, that the simulated clouds tend to mask rather than to enhance the notable discrepancies which exist between these clear-sky flux estimates in the various models (Wild 2008, 2019). This indicates that the downward longwave radiation from the cloud-free atmosphere is largely contributing to the spread noted in the (all-sky) downward longwave radiation across the various CMIP6 models.

Overall, under cloud-free conditions, the longwave budgets in the CMIP6 models still show substantial discrepancies and are not as consistently simulated as their shortwave counterparts, as reflected in considerably larger standard deviations and inter-model spreads (Table 1).

## Results—global cloud radiative effects

The quantification of both all-sky and clear-sky budgets allows an estimation of the effects that clouds exert globally on the energy flows in the various GCMs. In the following, the global cloud radiative effects (CRE) on the shortwave, longwave and net budgets are discussed as they apply at the TOA, within the atmosphere and at the Earth’s surface.

### TOA cloud radiative effects

**Fig. 10:**
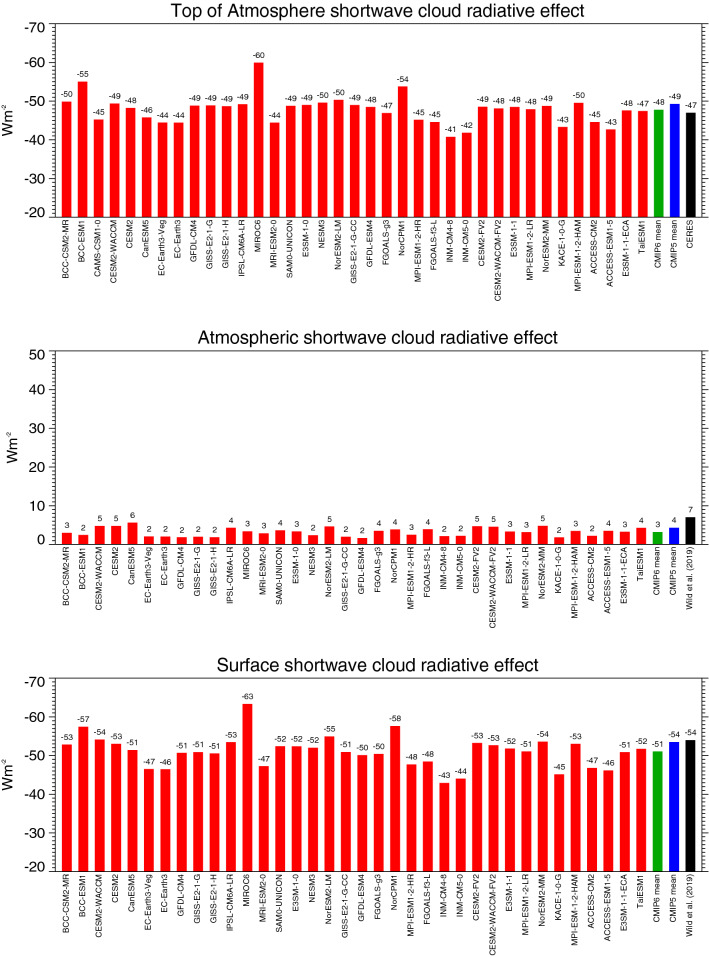
Global annual mean shortwave cloud radiative effects at the TOA (upper panel), within the atmosphere (middle panel) and at the surface (lower panel) representative for present-day climate, as simulated by various CMIP6 models (red bars). Cloud radiative effects determined as differences between the respective all-sky (Fig. 2) and clear-sky (Fig. 8) shortwave radiation budgets of the individual CMIP6 models. CMIP6 and CMIP5 multi-model means given by green and blue bars, respectively. Reference estimates from CERES (Loeb et al. 2018) and Wild et al. (2019) (black bars). Units Wm^−2^

The TOA shortwave absorption in the CMIP6 multi-model mean under clear-sky and all-sky conditions, at 287.3 and 239.5 Wm^−2^, respectively, differs by 47.8 Wm^−2^ globally. This implies that the overall effect of clouds in the CMIP6 models is to reduce the absorption of shortwave radiation in the climate system by – 47.8 Wm^−2^ (TOA shortwave CRE). This is in close agreement with the CERES EBAF reference estimate (Loeb et al. 2018), adjusted according to Method II for an exact comparison with climate models, of − 47 Wm^−2^ (Wild et al. 2019). However, the spread in the TOA shortwave CRE amongst the individual CMIP6 models is again substantial, ranging from − 41 to − 60 Wm^−2^ globally (Fig. 10 upper panel). This range is larger than in the CMIP5 models, despite the somewhat smaller number of models considered in CMIP6 (Table 1). Still two-third of the CMIP6 models simulate a global mean TOA shortwave CRE within 2 Wm^−2^ of the reference estimate.

**Fig. 11:**
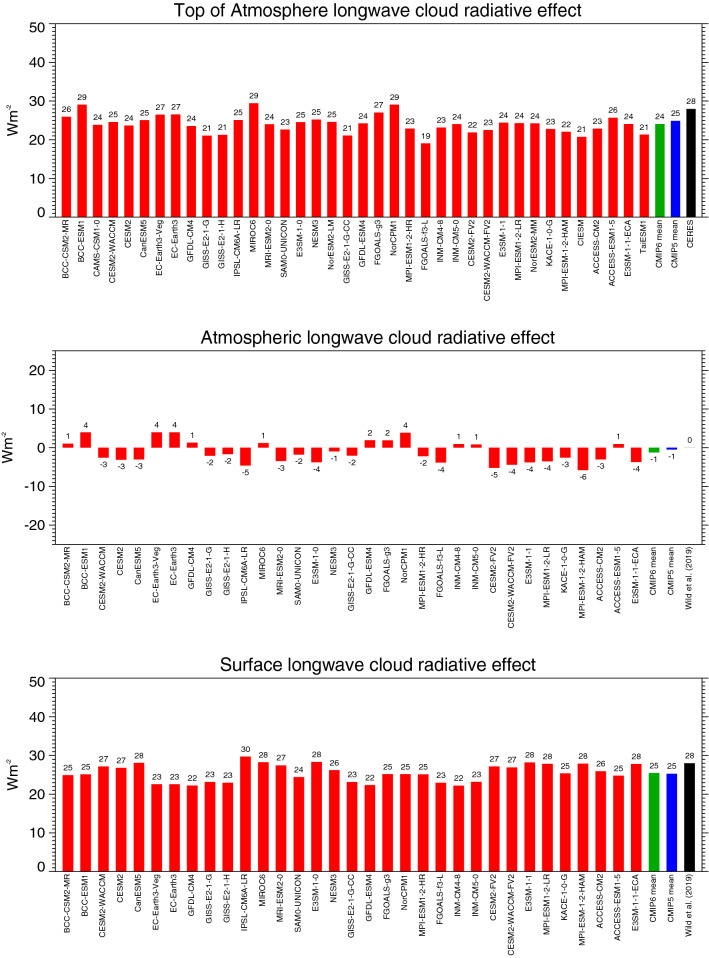
Global annual mean longwave cloud radiative effects at the TOA (upper panel), within the atmosphere (middle panel) and at the surface (lower panel) representative for present-day climate, as simulated by various CMIP6 models (red bars). Cloud radiative effects determined as differences between the respective all-sky (Fig. 4) and clear-sky (Fig. 9) longwave radiation budgets of the individual CMIP6 models. CMIP6 and CMIP5 multi-model means given by green and blue bars, respectively. Reference estimates from CERES (Loeb et al. 2018) and Wild et al. (2019) (black bars). Units Wm^−2^

Similarly, the difference between the global mean OLR under clear-sky and all-sky conditions in the CMIP6 multi-model mean, at − 262.4 Wm^−2^ and − 238.3 Wm^−2^, respectively, differs by 24.1 Wm^−2^. This implies that clouds globally reduce the longwave emission to space by 24.1 Wm^−2^ (TOA longwave CRE) in the CMIP6 multi-model mean, causing a gain of energy for the climate system of slightly lower amount than in the CMIP5 multi-model mean (Table 1, Fig. 11 upper panel). The TOA longwave CRE in both CMIP6 and CMIP5 multi-model means is weaker than in the CERES reference estimate adjusted for Method II (28 Wm^−2^, Table 1), due to the lower clear-sky OLR in the models as discussed in the previous section. The global mean TOA longwave CRE in the individual CMIP6 models ranges from 19 to 29 Wm^−2^ (Fig. 11 upper panel).

**Fig. 12:**
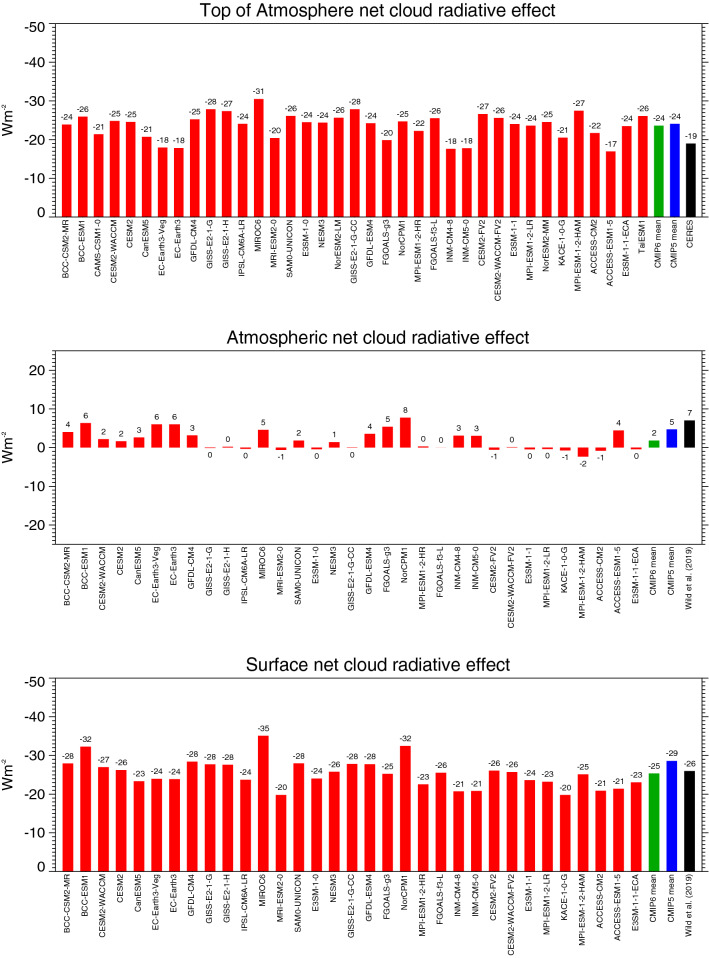
Global annual mean net (shortwave + longwave) cloud radiative effects at the TOA (upper panel), within the atmosphere (middle panel) and at the surface (lower panel) representative for present-day climate, as simulated by various CMIP6 models (red bars). Net cloud radiative effects defined as differences between the respective all-sky and clear-sky net radiation budgets of the individual CMIP6 models. CMIP6 and CMIP5 multi-model means given by green and blue bars, respectively. Reference estimates from CERES (Loeb et al. 2018) and Wild et al. (2019) (black bars). Units Wm^−2^

In terms of the net effect of clouds on the energy content of the climate system (TOA net CRE), the enhanced shortwave reflection of − 47.8 Wm^−2^ thus globally dominates over the longwave energy gain of 24.1 Wm^−2^ in the CMIP6 multi-model mean, which implies an overall energy reduction of − 23.7 Wm^−2^ for the climate system (TOA net CRE), close to the corresponding value of the CMIP5 multi-model mean (Table 1, Fig. 12 upper panel). This overall energy loss due to clouds is stronger than indicated in the corresponding CERES satellite reference estimates on the order of 5 Wm^−2^, primarily due to the weaker trapping of longwave outgoing radiation, plus a slightly stronger shortwave reflection back to space in the CMIP6 models (Table 1). The global mean TOA net CRE in the individual CMIP6 models ranges from − 17 to − 31 Wm^−2^ (Fig. 12 upper panel). Thus also most of the individual models simulate a more negative TOA net CRE than the reference estimates suggest.

### Atmospheric cloud radiative effects

The presence of clouds slightly enhances the shortwave absorption in the atmospheric column in all CMIP6 models (Fig. 10, middle panel). The CMIP6 multi-model mean atmospheric shortwave CRE is, at 3.2 Wm^−2^ globally, somewhat weaker than the CMIP5 multi-model mean estimate (statistically significant, Table 1).

The atmospheric cloud effect in the longwave is marginal in the CMIP6 multi-model mean, at -1.3 Wm^−2^ globally (Table 1), as in CMIP5. Individual CMIP6 model estimates vary in a range from − 6 to + 4 Wm^−2^ (Fig. 11, middle panel). This leaves a global mean net effect of clouds on the atmospheric column absorption of 1.9 Wm^−2^ in the CMIP6 multi-model global mean (3.6 Wm^−2^ in CMIP5, difference statistically significant, Table 1). The net effect of clouds is thus a slight enhancement of the atmospheric energy content globally. This slight enhancement is found in half of the individual CMIP6 models and reaches up to 8 Wm^−2^, while the other half shows a near zero effect or a slight reduction (Fig. 12 middle panel).

### surface cloud radiative effects

The effect of clouds on the absorption of shortwave radiation at the Earth’s surface (surface shortwave CRE) in the CMIP6 multi-model mean is a global mean reduction of − 51.2 Wm^−2^ (from 214.6 Wm^−2^ clear-sky absorption to 163.4 Wm^−2^ all-sky absorption). This magnitude falls within the reference estimates given in Table 1. The global mean surface shortwave CRE in the CMIP6 multi-model mean is weaker than in its CMIP5 counterpart (statistically significant, Table 1), due to the fact that the surface clear-sky shortwave absorption is more reduced than the all-sky absorption in the CMIP6 compared to the CMIP5 multi-model mean. Again the spread of the global estimates in the individual CMIP6 models is remarkable, covering a range of 20 Wm^−2^ (Fig. 10, bottom panel).

The effect of clouds on the longwave surface balance is to reduce the surface cooling by 25.5 Wm^−2^ globally in the CMIP6 multi-model mean, nearly matching its CMIP5 counterpart. This effect is somewhat smaller than the reference estimates indicate (Table 1), which are near to the upper bound of the individual model estimates given in Fig. 11 (bottom panel). Both spread and standard deviation in the surface longwave CRE of the CMIP6 models are substantially reduced compared to CMIP5.

As a net effect at the Earth’s surface (surface net CRE), the presence of clouds reduces the available energy by − 25.4 Wm^−2^ in the CMIP6 multi-model mean globally, since the energy gain for the surface in the longwave does not compensate the energy loss in the shortwave. The global mean surface net CRE is weaker in the multi-model mean in CMIP6 than in CMIP5 (statistically significant, Table 1), due to the weaker shortwave CRE as discussed above, and comes close to the reference estimate in Wild et al. (2019). The spread of the global mean surface net CRE in the individual CMIP6 models is illustrated in Fig. 12 (bottom panel).

## Discussion and conclusions

**Fig. 13:**
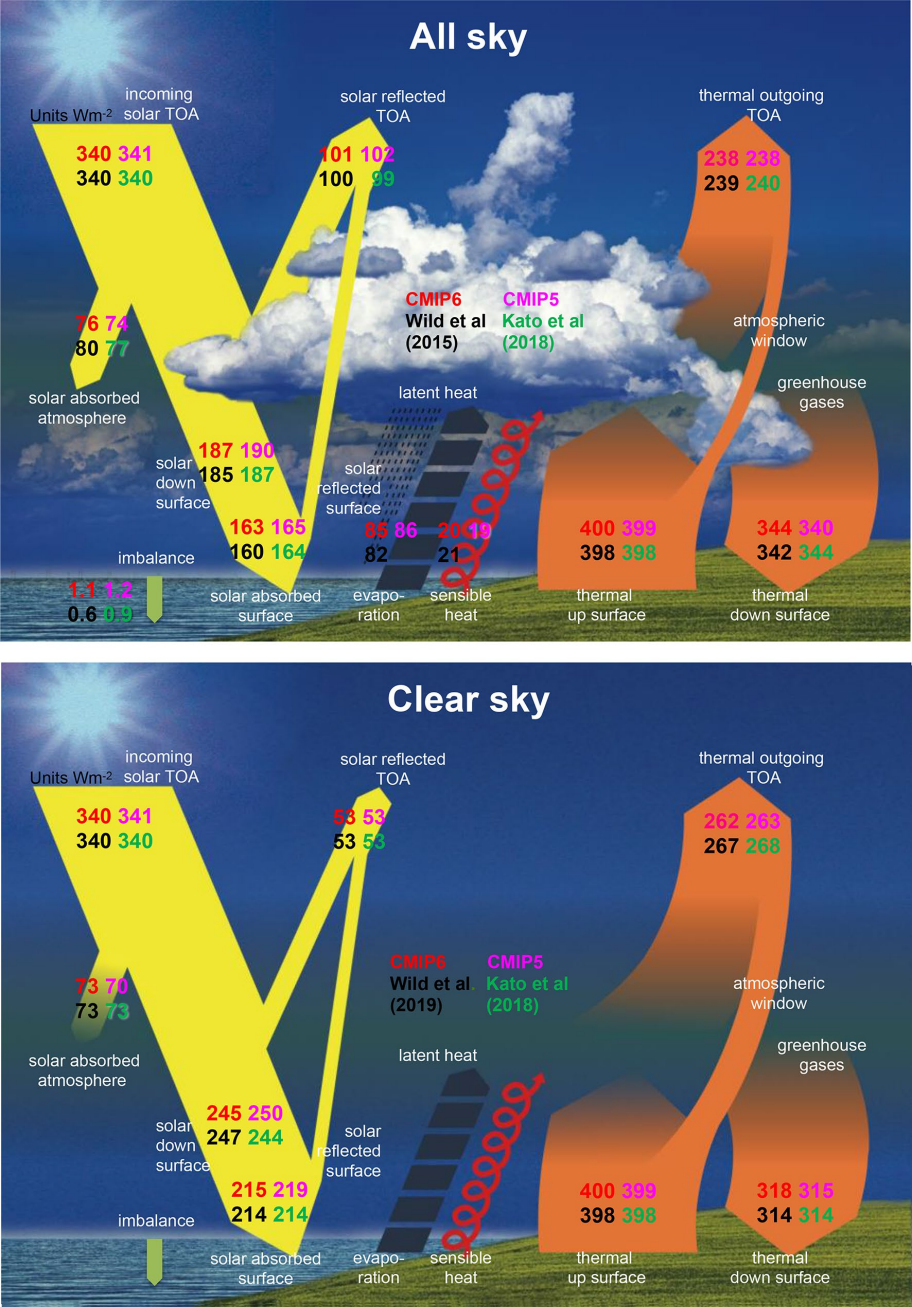
Comparison of different global annual mean energy balance estimates for present-day climate under “all-sky” (upper panel) and “clear-sky” (lower panel) conditions, as simulated in the CMIP6 multi-model mean (upper left (red) values) and in the CMIP5 multi-model mean (upper right (pink) values), and as estimated by Wild et al. (2015, 2019) (lower left (black) values) and Kato et al. (2018) (lower right (green) values). Values attached to arrows correspond to energy fluxes in Wm^−2^ in the direction given by the arrows. Averaging periods for CMIP5 and Wild et al. (2015, 2019): 2000–2004; CMIP6: 2000–2014; Kato et al. (2018): 2005–2015

The global energy budget components of up to 40 newly available GCMs participating in CMIP6 have been assessed both under all-sky and clear-sky conditions, covering TOA, surface and atmospheric budgets. On a global multi-model mean basis, the simulated energy balance components in CMIP6 are in the majority close to recent reference estimates, often closer than any preceding model generation, and particularly close in case of the shortwave clear-sky budgets. This is also evident from Fig. 13, which summarizes the CMIP6 and CMIP5 multi-model mean magnitudes of the various global energy balance components in graphical form and compares them with two recent reference estimates. The good agreement of the CMIP6 multi-model means with the reference estimates is not only evident in the TOA components where the reference estimates are commonly used as tuning targets, but increasingly also in other quantities not directly considered in the tuning process (Fig. 13). Note that this does not necessarily apply for the individual CMIP6 models. Despite the tuning efforts applied in model development to match particularly the simulated TOA global mean fluxes with the observational space-based references, 9 (8) CMIP6 models still simulate a global mean shortwave TOA absorption (OLR) outside the 2-sigma observational uncertainty given in Loeb et al. (2009).

In terms of the surface energy budget, a prominent and persistent model bias consisted for many years in a too large shortwave irradiance at the Earth’s surface, which was partly compensated by a overly small downward longwave radiation, leading to a superficially correct surface net radiation in the global mean due to this error cancellation, an issue noted already back in the 1990s (Wild et al. 1995). This excessive insolation and compensational lack of downward longwave radiation has not only been found under all-sky conditions, but similarly also under clear-skies (Wild et al. 1995, 2006; Wild 2008). The excessive surface insolation has therefore been related to a lack of absorption in the cloud-free atmosphere in the models. It is interesting to note that the amount of shortwave radiation absorbed within the cloud-free atmosphere under present-day conditions as simulated by climate models has been gradually adjusted upwards from one model generation to the next during the history of GCM development. This is documented in Table 2, which shows the evolution of multi-model global means of shortwave absorption in the cloud-free atmosphere over several generations of GCMs, from early models representing the status in the late 1980s/early 1990s, up to the most recent model generation CMIP6. The model-representation of shortwave absorption in the cloud-free atmosphere increased during this development process on the order of 10 Wm^−2^ (15% of its absolute value), thereby contributing to counteract the excessive surface insolation bias. This upward adjustment brings the shortwave absorption in the cloud-free atmosphere of the CMIP6 multi-model mean now also in close agreement with the recent independently derived reference estimates of Kato et al. (2018) and Wild et al. (2019) of 73 Wm^−2^, also given in Table 2 and Fig. 13 for comparison. Another independent reference estimate amounts to 72 Wm^−2^ based on a combination of global satellite-derived data sets for aerosols, water vapor and total ozone and a Monte Carlo Aerosol-Cloud-Radiation (MACR) model (Kim and Ramanathan 2008), and thus gives further quantitative support for the magnitudes of the above reference estimates. It is also remarkable that the global mean shortwave absorption in the cloud-free atmosphere simulated by the CMIP6 models is not only close to these recent reference estimates in their multi-model mean, but also in the individual models, most of them deviating less than 2 Wm^−2^ from the reference estimates (see Sect. 4.1). The gradual upward adjustment in the simulated present-day shortwave absorption in the cloud-free atmosphere over the history of model development has been favored by the inclusion of absorbing aerosol in the radiation codes of the models [the early models did only consider sulfur-based scattering aerosols, or did not consider aerosols at all, e.g., Cusack et al. (1998)]. Also, atmospheric water vapor absorption has been underestimated by the early radiation codes, and has increased during the evolution of model development, based on newer assessments of the spectroscopic absorption coefficients and improved formulations of the near-infrared water vapor continuum (Wild et al. 1998; Morcrette 2002; Pincus et al. 2015; Paynter and Ramaswamy 2012; Radel et al. 2015; Paynter and Ramaswamy 2014). This has also been noted in the Continual Intercomparison of Radiation Codes (CIRC, Oreopoulos and Mlawer 2010; Oreopoulos et al. 2012) as well as in preceding radiation code intercomparison projects (Fouquart et al. 1991; Barker et al. 2003). Therein also some missing, yet well-established radiation physics, such as the neglection of N_2_O and CH_4_ absorption in some of the earlier radiation codes has been identified (Collins et al. 2006), which has been taken into account in the meantime in modern radiation codes.

**Table 2:** Historic evolution of the quantitative representation of present-day global annual mean shortwave atmospheric absorption under clear-sky conditions in multi-model means of different generations of climate models covering 30 years of model development

Model Generation	# of models	Multi-model mean (Wm^−2^)	References
Pre-AMIP (late 1980s)	7	63	Wild et al. (1998)
AMIPII (1990s)	20	67	Wild et al. (2006)
CMIP3 (early 2000s)	14	69	Wild et al. (2006)
CMIP5 (late 2000s)	43	70	Wild et al. (2019)
CMIP6 (late 2010s)	36	73	This study
Recent reference estimates		73	Wild et al. (2019)
		73	Kato et al. (2018)
		72	Kim and Ramanathan (2008)

For comparison also recent reference estimates are added

Units Wm^−2^

Another persistent issue in the model-calculated surface energy budgets over the history of GCM model development has been the abovementioned underestimation of downward longwave radiation when compared to surface observations, as we first noted in Wild et al. (1995). Uncertainties in the formulation of the water vapor continuum have been contributing to this underestimation (Iacono et al. 2000; Wild et al. 2001). During the course of model development over the past 30 years, the simulated present-day downward longwave radiation has overall been gradually adjusted upwards from one model generation to the next, as indicated in Table 3. Thereby, considerable progress has been made in reducing these biases during the course of model developments (Ma et al. 2014; Wild et al. 2015, 2019). Note that the early model generations are representative of a slightly earlier period (1980s/1990s) than the one used for CMIP5 and CMIP6 (early 2000s), and thus are expected to have a slightly smaller downward longwave radiation due to the somewhat weaker greenhouse forcing in the earlier period. However, this effect can only account for a minor fraction of the differences in the downward longwave radiation between the different model generations. The multi-model global mean downward longwave radiation in the CMIP6 models, at 343.8 Wm^−2^, is now in near perfect agreement with recent independent reference estimates, also given in Table 3. Note that the slightly lower reference value given in Wild et al. (2013, 2015), at 342 Wm^−2^, is derived for the period 2000–2004, which converted to the model analysis period 2000–2014 would be higher by about 0.8 Wm^−2^ due to somewhat stronger greenhouse forcing and warming on average over this period (see Sect. 2), and thus even closer to the CMIP6 multi-model mean.

**Table 3:** Historic evolution of the quantitative representation of present-day global annual mean downward longwave radiation in multi-model means of different generations of climate models covering 30 years of model development

Model Generation	# Of models	Multi-model mean (W m^−2^)	References
Pre-AMIP (late 1980s)	6	327	Wild et al. (1995)
	11	329	Wild et al. (2001)
AMIPII (1990s)	20	336	Wild (2008)
CMIP3 (early 2000s)	20	337	Wild (2008)
CMIP5 (late 2000s)	22	338	Wild et al. (2013)
	43	340	Wild et al. (2015)
CMIP6 (late 2010s)	38	344	This study
Recent reference estimates		342	Wild et al. (2013, 2015)
		342	Wang and Dickinson (2013)
		341	L’Ecuyer et al. (2015)
		344	Kato et al. (2018)

Therefore, the long-standing tendency in the present-day GCM surface energy budgets to compensate an excessive surface shortwave radiation with a too small downward longwave radiation globally, is now to a large degree remediated in the CMIP6 multi-model mean.

While the global surface radiation budget in the CMIP6 multi-model mean seems now to be quite realistic, and probably more realistic in terms of its multi-model mean than in any preceding model generation, further development work needs to be done by some of the individual modelling groups to converge to this level as well. Indeed the inter-model spread amongst the magnitudes of the global energy balance components in the individual CMIP6 models is still unsatisfactorily large, typically on the order of 10–20 Wm^−2^. The substantial inter-model spread of 18 Wm^−2^ in the simulated global mean surface latent heat flux further points to considerable discrepancies not only in the representation  of the global energy cycle, but also of the global water cycle in the CMIP6 models. All these discrepancies have generally not decreased from the previous model generation CMIP5 to the latest model generation CMIP6, and the inter-model spreads and standard deviations remain similar. Thus, there is no clear sign of convergence in the energy budget estimates of current state-of the art climate models. An exception state the clear-sky shortwave budgets, which are now not only similarly represented in the majority of the CMIP6 models in terms of their global means, but also closely match recent reference estimates.

The substantial discrepancies in the representation of some of the energy balance components between the various CMIP6 models noted here on a global annual mean basis are worrisome as the inter-model spread will undoubtedly further increase on regional, seasonal and diurnal scales. This has major implications for the simulation of regional climates, which cannot be excepted to reach a high degree of consistency amongst the different models under these conditions. Convergence in the representation of the energy budgets by the various models on a global mean basis is therefore a necessary, but not sufficient prerequisite for consistent simulations of regional energy budgets and climates.
